# Confirming a critical role for death receptor 5 and caspase-8 in apoptosis induction by endoplasmic reticulum stress

**DOI:** 10.1038/s41418-018-0155-y

**Published:** 2018-07-10

**Authors:** Mable Lam, David A. Lawrence, Avi Ashkenazi, Peter Walter

**Affiliations:** 1 Howard Hughes Medical Institute, Department of Biochemistry and Biophysics, University of California at San Francisco, San Francisco, CA 94158 USA; 2 Cancer Immunology, Genentech, Inc., South San Francisco, CA 94080 USA

## Abstract

Several studies implicate specific death receptors (DRs) and caspase-8 in mediating apoptosis in response to endoplasmic reticulum (ER) stress; however, a recent paper challenges this conclusion. Here we validate the importance of DR5 and caspase-8 as critical signal conduits for apoptosis activation upon ER stress.

Elucidating the switch between cell survival and apoptosis during ER stress is key to understanding diseases such as protein-folding disorders and cancer [1, 2]. Several reports demonstrate a critical role for DRs and caspase-8 in ER stress-induced apoptosis [3–7]. However, Glab et al. [8] recently refuted this by studying cell lines bearing CRISPR knockout (KO) of DR5 or caspase-8. Glab et al. kindly provided their cells to us upon request, and we have analyzed these independently at both of our laboratories. Here we confirm, using their cell lines, that DR5 and caspase-8 have crucial roles in triggering apoptosis upon in response to ER stress [8].

We induced ER stress with thapsigargin (Tg) and measured apoptosis activation by two orthogonal assays: Annexin V/Sytox Blue staining (Fig. S1A–D) and subG1 DNA content (Fig. S1E). Relative to HCT116 Cas9 parental cells, DR5 KO cells showed partial attenuation, while caspase-8 KO cells exhibited nearly complete inhibition of Tg-induced apoptosis. Consistently, Tg-induced PARP cleavage (evident by the ratio of cleaved to uncleaved PARP, quantified in Fig. S1G), caspase-3 cleavage, and caspase-3/7 enzymatic activity, were diminished in DR5 KO cells and abolished in caspase-8 KO cells (Fig. S1F–H).

We also performed siRNA knockdown against DR5 in Cas9 parental cells. In keeping with published data, DR5 depletion markedly inhibited Tg-induced apoptosis, as clearly indicated by reduced Annexin V/Sytox Blue staining, PARP and caspase-3 cleavage, and caspase-3/7 activity (Fig. S2A–F). Transfection of DR5 KO cells with DR5 siRNA had no further effect on Tg-induced apoptosis, verifying an on-target impact of DR5 siRNA in the Cas9 parental cells (Fig. S3A–C). Of note, DR5 CRISPR KO suppressed apoptosis less effectively than did DR5 siRNA depletion (compare Figs. S1 and S2). Furthermore, although caspase-8 KO in Cas9 parental cells strongly inhibited apoptosis, caspase-8 siRNA depletion in DR5 KO cells did not (Fig. S3D, E), suggesting that the residual, DR5-independent, apoptosis in DR5 KO cells is also caspase-8 independent. A plausible explanation of these findings is that long-term DR5 ablation by CRISPR has caused the selected cell line to adapt a rewired ER-stress-responsive apoptotic pathway. Caspase-8 KO cells did not display this, perhaps because this enzyme funnels more apoptosis drivers and is therefore more difficult to bypass than DR5. Why we find caspase-8 KO cells to be resistant whereas Glab et al. found these to be sensitive to ER stress awaits further investigation.

Inhibition of Tg-induced apoptosis was also observed upon TALEN-edited KO of DR5 and DR4 [6]. The robust apoptosis inhibition upon acute DR5 depletion and genetic caspase-8 ablation verified herein demonstrates that the DR pathway is a critical conduit for life-vs-death decisions during ER stress. This principle has now been documented for diverse ER stressors across multiple cell types [9]. In keeping with this conclusion, both our published results [3] and those of Glab et al. [8] implicate the BH3-only protein Bid, whose key proteolytic trigger is caspase-8, in ER stress-induced apoptosis.

## Electronic supplementary material

Genetic ablation of DR5 partially attenuates, while disruption of caspase-8 completely blocks, Tg-induced apoptosis

Acute depletion of DR5 via siRNA knockdown substantially inhibits ER stress-induced apoptosis

Apoptosis in DR5 KO cells occurs independently of caspase-8 Exchange references 7 and 8 below such that Yamaguchi et al is 7 and Glab et al is 8

## Electronic supplementary material

Note that Figure 1 needs to be replaced with attached fileThe online version of this article (10.1038/s41418-018-0155-y) contains supplementary material, which is available to authorized users.
