# Supplementary material for: Confirming a critical role for death receptor 5 and caspase-8 in apoptosis induction by endoplasmic reticulum stress
Source: Cell Death Differ. 2018 Jul 10;25(8):1530–1. doi: 10.1038/s41418-018-0155-y (PMC6113221; doi:10.1038/s41418-018-0155-y)
Supplement: Supplementary file 1 — Genetic ablation of DR5 partially attenuates, while disruption of caspase-8 completely blocks, Tg-induced apoptosis [file 41418_2018_155_MOESM1_ESM.pdf]

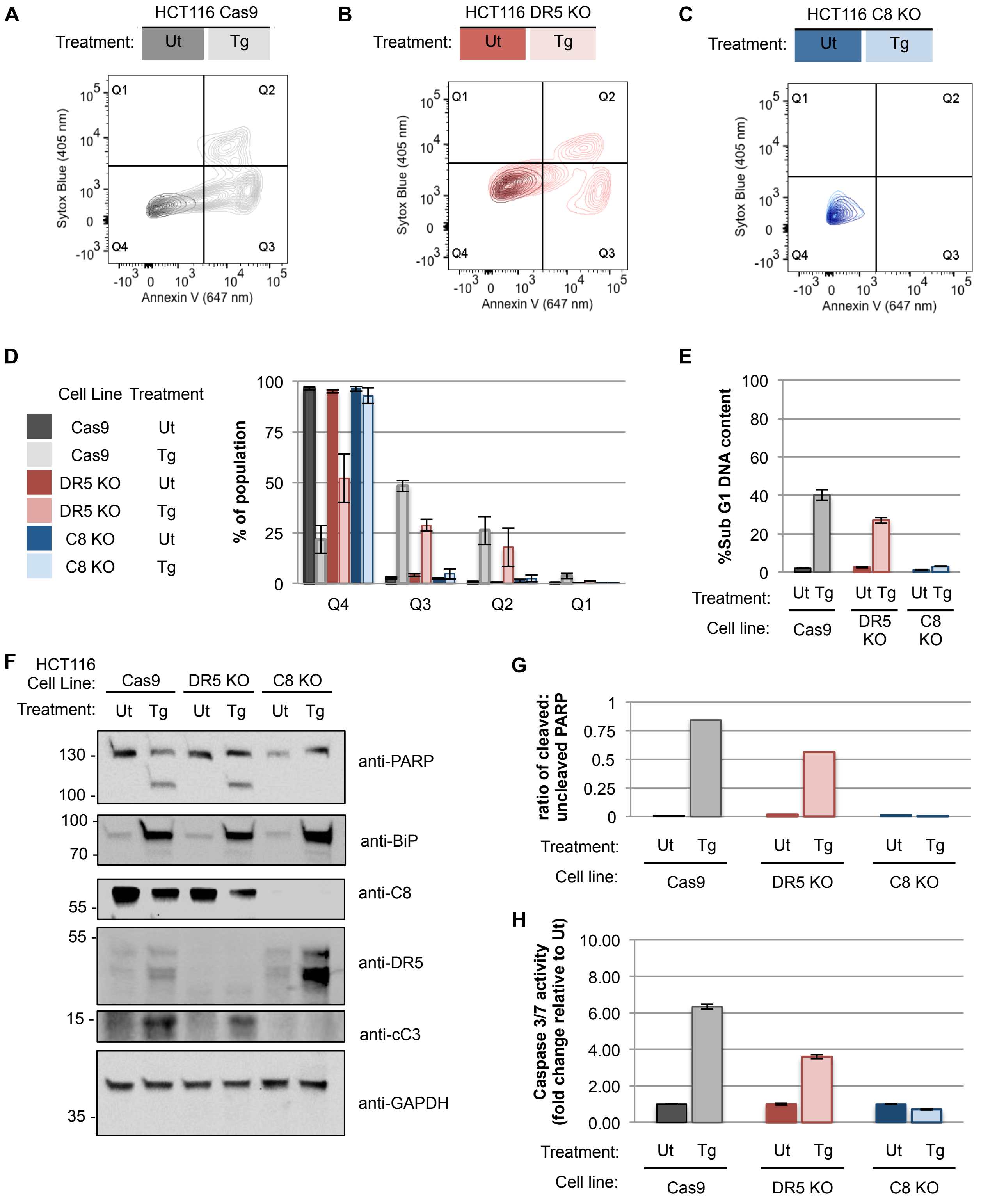

Figure S1: Genetic ablation of DR5 partially attenuates, while disruption of caspase 8 completely blocks, Tg-induced apoptosis.

HCT116 Cas9 parental, DR5 KO cells, or caspase-8 KO cells were treated with thapsigargin (Tg, 100 nM) or vehicle alone (Ut) for 24 hours. Cells were then stained with Annexin V-AlexaFluor647 and Sytox Blue Dead Cell stain to quantify the percentage of live (Q4), early apoptotic (Q3), and late apoptotic (Q2) cells via flow cytometry for three biological replicates, each containing 10000-30000 cells (A-D). Apoptosis was also measured by analyzing subG1 DNA content via flow cytometry (E, mean -/+ standard deviation for n = 2). Lysates of DR5 KO and caspase 8 KO cells were probed for PARP cleavage and caspase 3/7 activity by Western blotting (F) and GLO assay (H, mean -/+ standard deviation for n = 3 biological replicates). The ratio of cleaved to uncleaved PARP was quantified using ImageJ to measure the intensity of the bands at 130 and 100 kDa in the anti-PARP blot (G).
