# Supplementary material for: Confirming a critical role for death receptor 5 and caspase-8 in apoptosis induction by endoplasmic reticulum stress
Source: Cell Death Differ. 2018 Jul 10;25(8):1530–1. doi: 10.1038/s41418-018-0155-y (PMC6113221; doi:10.1038/s41418-018-0155-y)
Supplement: Supplementary file 2 — Acute depletion of DR5 via siRNA knockdown substantially inhibits ER stress-induced apoptosis [file 41418_2018_155_MOESM2_ESM.pdf]

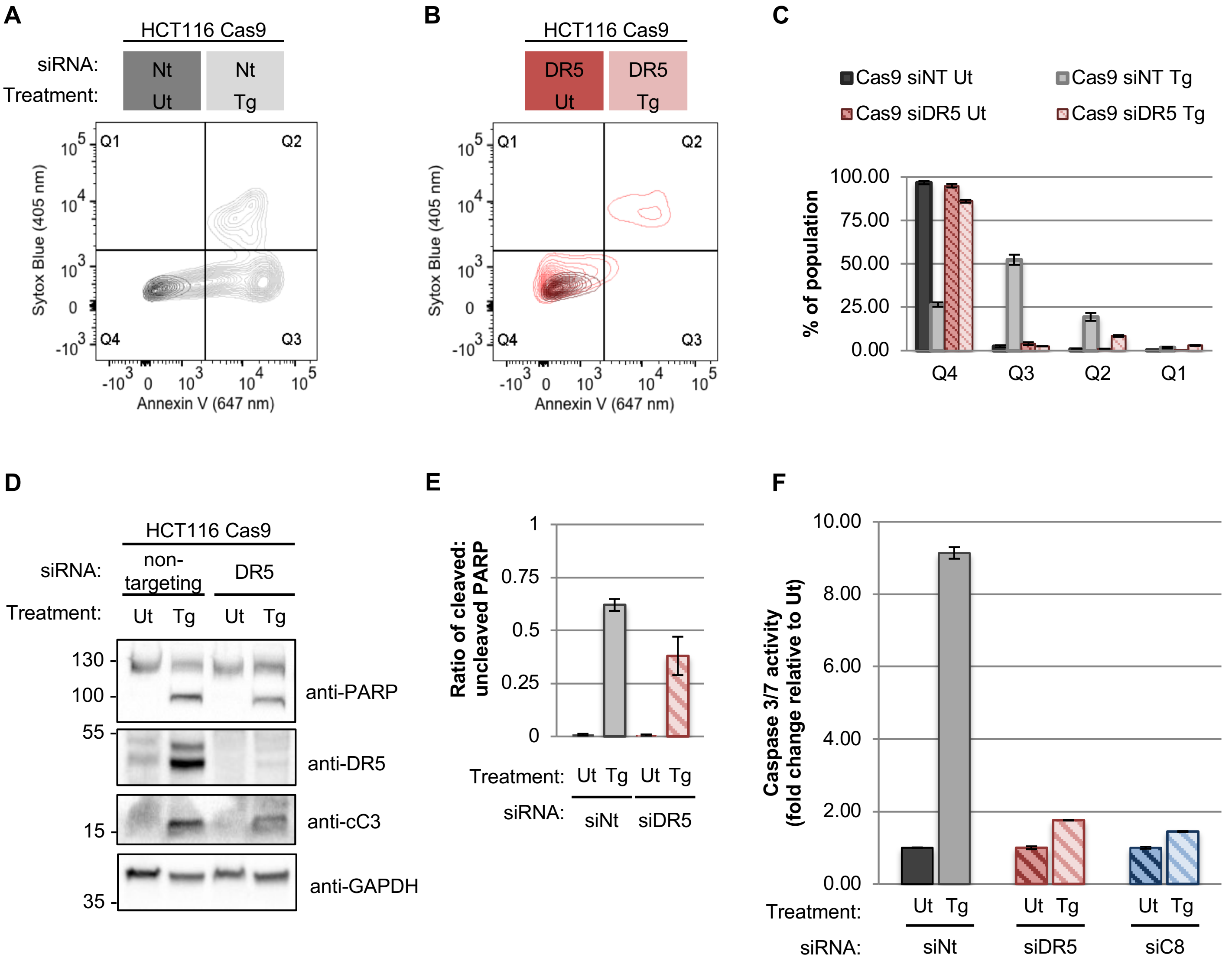

Figure S2: Acute depletion of DR5 via siRNA knockdown substantially inhibits ER stress-induced apoptosis.

HCT116 Cas9 parental cells were transfected with siRNA against DR5 or a non-targeting control (Nt) for 48 hours prior to treatment with 100 nM Tg for 24 hours. Cells were then stained with Annexin V-AlexaFluor647 and SytoxBlue Dead cell stain to quantify the population of live (Q4), early apoptotic (Q3), and late apoptotic (Q2) through flow cytometry (A-C). Lysates of Cas9 parental cells treated with siRNA against DR5 were analyzed for PARP cleavage via Western blotting, where one representative replicate is shown, (D) and caspase-3/7 activity through GLO assay (F, mean -/+ standard deviation for n = 3 biological replicates). The ratio of cleaved to uncleaved PARP was quantified using ImageJ to measure the intensity of the bands at 130 and 100 kDa in the anti-PARP blot (E, mean -/+ standard deviation for n = 3 biological replicates).
