# Supplementary material for: Confirming a critical role for death receptor 5 and caspase-8 in apoptosis induction by endoplasmic reticulum stress
Source: Cell Death Differ. 2018 Jul 10;25(8):1530–1. doi: 10.1038/s41418-018-0155-y (PMC6113221; doi:10.1038/s41418-018-0155-y)
Supplement: Supplementary file 3 — Apoptosis in DR5 KO cells occurs independently of caspase-8 Exchange references 7 and 8 below such that Yamaguchi et al is 7 and Glab et al is 8 [file 41418_2018_155_MOESM3_ESM.pdf]

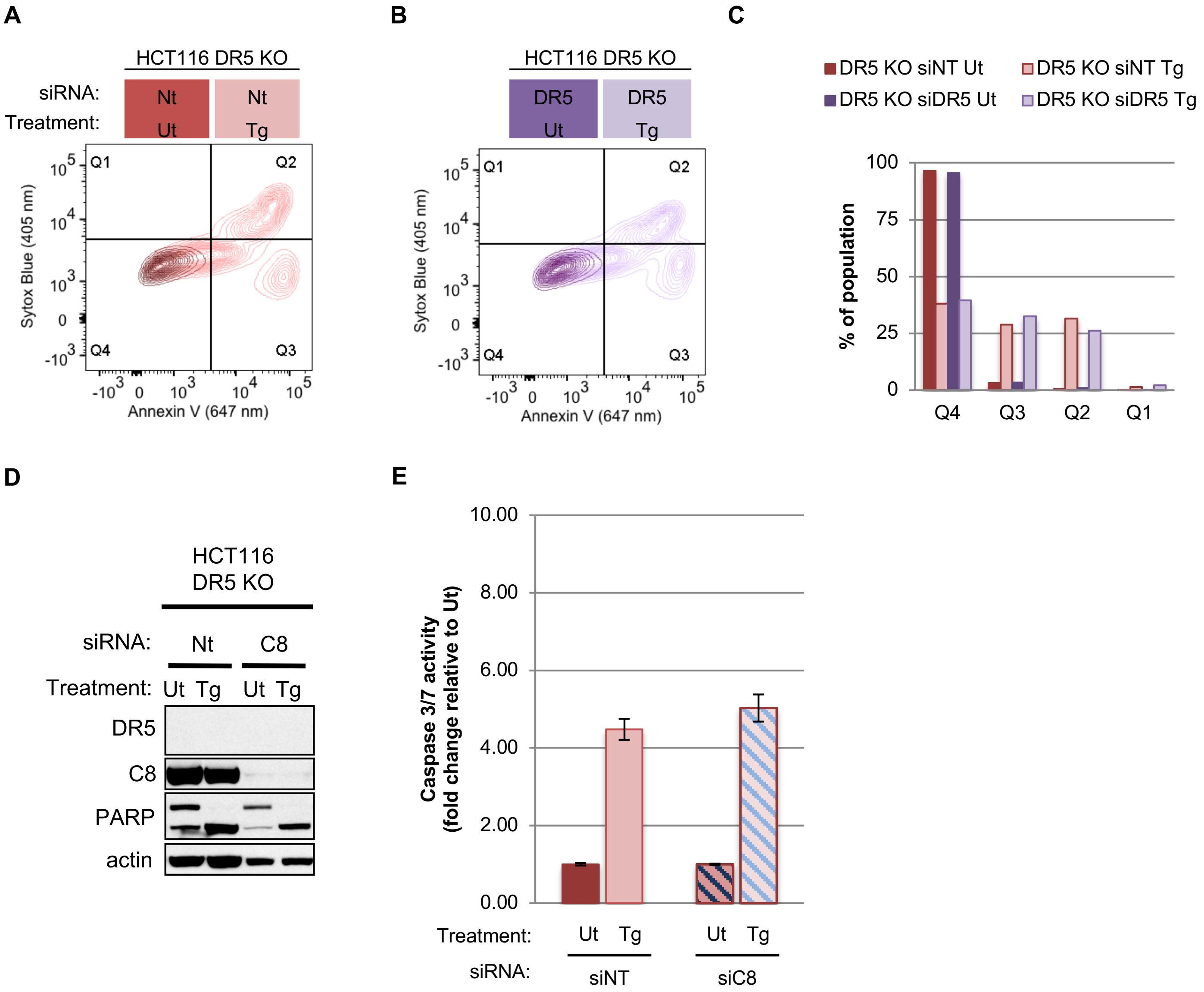

Figure S3: Apoptosis in DR5 KO cells occurs independently of caspase-8.

Apoptosis was measured via Annexin V-Sytox Blue staining for HCT116 DR5 KO cells transfected with siRNA against DR5 or non-targeting control (Nt) for 48 hours and treated with 100 nM Tg or vehicle alone (Ut) for 24 hours (A-C). DR5 KO cells transfected with siRNA against caspase-8 were analyzed for PARP cleavage via Western blotting (D) and for caspase-3/7 activity via GLO assay (E, mean -/+ standard deviation for n = 3 biological replicates).
